# A New Electrochemical Sensor for Direct Detection of Purine Antimetabolites and DNA Degradation

**DOI:** 10.1155/2019/1572526

**Published:** 2019-03-07

**Authors:** Liliya K. Shpigun, Elena Yu. Andryukhina

**Affiliations:** Institute of General & Inorganic Chemistry of Russian Academy of Sciences, 119991 Moscow, Russia

## Abstract

The development of a reliable electrochemical sensor using a hybrid nanocomposite consisting of ionic liquid (1-butyl-3-methylimidazolium hexafluorophosphate) functionalized graphene oxide (GrO-IL) and gold nanoparticles (AuNPs) stabilized by chitosan (Chit) was described. The new sensor, labelled as GrO-IL-AuNPs-Chit/CSE, exhibited an improved electrocatalytic response to cancer drugs such as purine antimetabolites (6-thioguanine, 6-mercaptopurine, and azathioprine) in a wide concentration range with a low detection limit (20–40 nmol·L^−1^, S/N = 3), and satisfactory recoveries (97.1–103.0%). The sensor has been also successfully used for cyclic voltammetric study of a salmon sperm double-stranded DNA degradation and DNA-6-mercaptopurine interaction in aqueous solutions (pH 7.4).

## 1. Introduction

The family of purines and purine analogs is the most common class of nitrogen-containing heterocyclic compounds which play crucial role in a wide variety of functions of living organisms. In particular, purine bases like adenine (9*H*-purin-6-amine, Ade) and guanine (2-amino-9*H*-purin-6(1*H*)-one, Gua) are components of nucleosides, the building blocks of DNA and RNA [1]. The concentration changes of Gua and Ade and their ratio in the DNA could be considered as important indicators in the clinical diagnosis and treatment of various diseases [2]. The purine antimetabolites such as 6-thioguanine (2-amino-1H-purine-6(7H)-thione, TG), 6-mercaptopurine (3,7-dihydro-purine-6-thione, MP), and azathioprine (6-([(1-methyl-4-nitro-1H-imidazol-5-yl)-sulphonyl]-7H-purine, AZTP) are commonly used in oral chemotherapy for the treatment of leukemia and became increasably popular in therapy of inflammatory diseases (ulcerative colitis, dermatitis, and some other pathologies) [3–5]. The cytostatic effect of all these compounds is associated with impaired synthesis of nucleic acids (DNA and RNA). The action mechanism of thiopurine drugs is probably due to the fact that they may interfere with the DNA synthesis and inhibit the proliferation of quickly growing cells, especially cells of the immune system, after the metabolic conversion to thiopurine nucleotides that substitute regular Ade and Gua nucleotides [6]. Not coincidentally, the chemistry of purine antimetabolites is still one of the most important areas of scientific research. Among the known publications on this topic, a lot of analytical techniques such as HPLC/LC [7–16], CE [17–19], FIA [20, 21], and SIA [22] have been proposed for the determination of these substances in pharmaceutical and clinical samples. The spectrophotometric, spectrofluorimetric, or chemiluminescence detection was based on the oxidation of an imidazole ring in their molecules by typical oxidizing agents [23–28]. The redox activity of purine antimetabolites determined the possibility of their electrochemical detection by using (bio)sensors based on carbon nanotubes-modified materials [29–38]. In recent years, a few studies have been reported, in which graphene- (Gr-) or graphene oxide- (GrO-) based nanocomposites were proposed as electrochemical sensing materials for thiopurines [39–43]. For instance, AZTP sensors were fabricated by electrodeposition of Gr-Chit composite onto the GCE surface [39] and by modification of a graphite electrode by Gr nanosheets decorated with Ag nanoparticles [40]. The development of a pencil graphite electrode modified with poly(neutral red)-electrochemically reduced GrO composite was described for sensing TG in biological and pharmaceutical samples [41]. In another paper [42], the voltammetric behaviour of TG was comparatively investigated on GrO- and reduced GrO-modified carbon paste electrodes. The voltammetric determination of MP at Co(III) tris-phenanthroline complex immobilized on a GCE modified with GrO-decorated DNA was described [43]. In addition, reduced GrO-modified electrode was fabricated to investigate the electrochemical oxidation of nucleic acids [44]. Recently, we proposed the electrochemical sensor based on the GrO-IL nanocomposite immobilized into a Chit biopolymeric matrix [45]. The major aim of the present study was to prepare a more sensitive and reliable electrode material, considering integration of the unique properties of GrO-IL and electrocatalytic activity of gold nanopieces- (AuNPs-) Chit bioconjugate. According to our knowledge, this concept has not been explored to achieve better electrochemical properties. The analytical perspectives and applicability of the new sensor for the voltammetric analysis of thiopurine anticancer drugs and for probing the double-stranded deoxyribonucleic acid (ds-DNA) damage were evaluated.

## 2. Experimental

### 2.1. Reagents and Solutions

GrO (powder), medium molecular weight сhitosan (75–85% deacetylation), HAuCl_4_·3H_2_O (99%), 1-butyl-3-methylimidazolium hexafluorophosphate ([BMIM]PF_6_), purines and thiopurine drugs (guanine, adenine, azathioprine, 6-thioguanine, and 6-mercaptopurine monohydrate), double-stranded DNA (ds-DNA) from salmon sperm (31149-11G-F), and other chemicals were purchased from Sigma-Aldrich Chemical Co. All compounds were of analytical grade and used as received. Stock solutions of each purine (1.0 mmol·L^−1^) were prepared by dissolving in 5.0 mmol·L^−1^ NaOH. Stock solution of native ds-DNA was prepared by dissolving 0.1 g of the sample in 100 mL water. All stock solutions were stored at +4°C in the dark at least one week. Working standard solutions were freshly prepared by stepwise diluting the respective stock solutions with a 0.1 mol·L^−1^ phosphate buffer solution containing 1.0 mol·L^−1^ KCl. A chitosan solution (0.5%) was prepared by dissolving 50 mg of Chit in 10 mL 1.0% (v/v) acetic acid. The solutions were deoxygenated by passing nitrogen gas. Hyperpure water was used throughout the experiment.

### 2.2. Apparatus

All electrochemical experiments were performed using an Ecotest-VA analyser (Econix-Expert, Russia) interfaced to a computer system with MDEV software. A three-electrode system was used, where a modified carbositall Dick electrode (CSE, Volta, Russia, diameter of 3 mm) served as the working electrode, an Ag/AgCl (3 mol·L^−1^ KCl) served as the reference electrode, and a platinum wire served as the auxiliary electrode. All potentials reported were referred to the Ag/AgCl electrode at 25 ± 1°C. The computer program Origin 2017 based on the Levenberg–Marquardt algorithm was used for signal processing and peak analysis. pH values were tested by using a pH-meter Model OP-110 (Radelkis, Hungary). The ultrasonic bath (Elmasonic One, Germany, 35 kHz ultrasound) was used in all ultrasonic experiments. The source of UV light was a PRO-4 lamp with a power of 4 W. UV-Vis absorbance spectra of the prepared chitosan-stabilized gold solutions were obtained using a JENWAY 6705 spectrophotometer (Bibby Scientific Ltd.). The measurements were carried out using a quartz cell of thickness 1 cm in the wavelength range of 200–700 nm. The surface morphology of GrO and the prepared composites was examined by using a scanning electron microscope (SEM, Carl Zeiss NVision 40, Germany).

### 2.3. Sensor Design

**Scheme 1:**
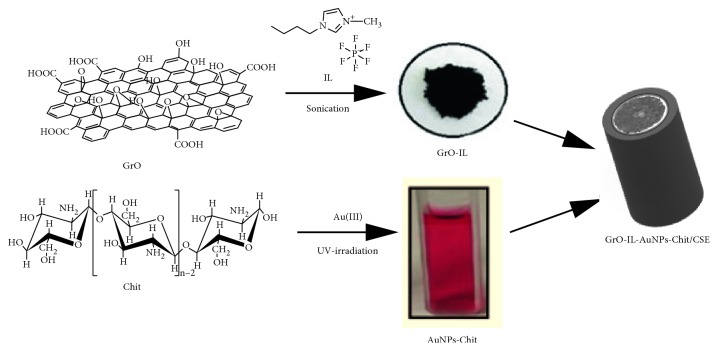
Schematic illustration of the fabrication process for GrO-IL-AuNPs-Chit/CSE.

The fabrication process for the sensor is depicted in Scheme 1.

**Figure 1:**
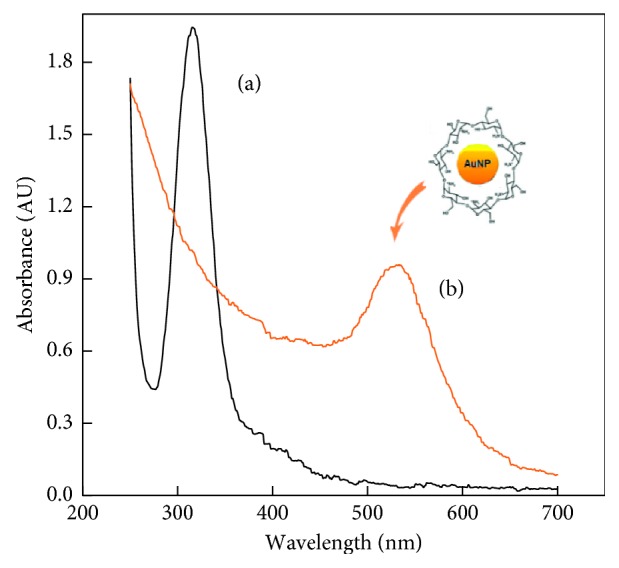
Absorbance spectra of a HAuCl_4_-Chit solution obtained before (a) and after (b) UV irradiation.

The hybrid nanocomposite GrO-IL-AuNPs-Chit was prepared in three steps. In the first step, the biogenic synthesis of AuNPs stabilized by Chit was carried out by UV-induced reduction of Au(III) to Au(0) in an acidic solution of Chit, as follows [46]: 1 mL of 2.0 mmol·L^−1^ HAuCl_4_ was added to 5 mL of 1.0 wt.% sodium citrate solution, and the mixture was heated to 50°C with constant stirring. Next, 2 mL of this mixture was added to 2.0 mL of the Chit solution (5 mg·mL^−1^), and the resulting solution was subjected to UV exposure until its colour turned rose-red. The time of UV irradiation was 50 min.  Figure 1 displays the UV-Vis spectra of a HAuCl_4_–Chit solution before and after UV irradiation.

The final spectra indicated that intensive gold reduction occurred during the UV irradiation, as it evidenced by the disappearance of the 315 nm band and the appearance of a new relatively broad band with a maximum of 530 nm, which is due to surface plasmon resonance for metal particles having an average size of about 5 nm [47, 48]. The resulting bioconjugate was labelled as AuNPs-Chit.

**Figure 2:**
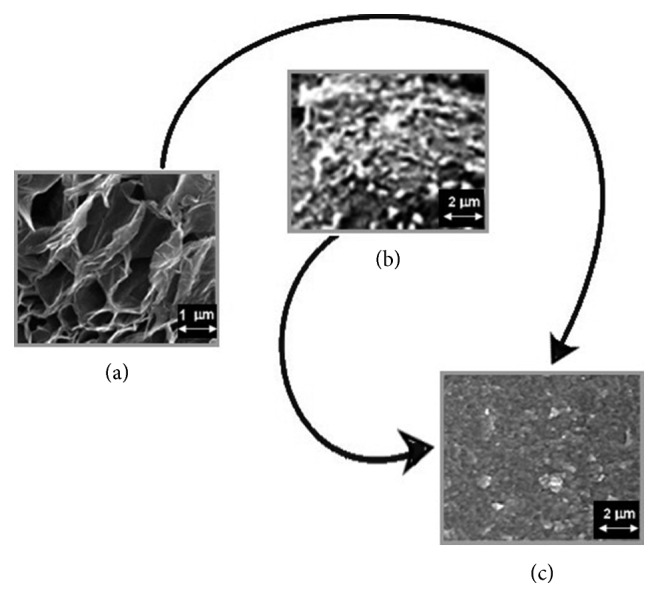
SEM images of the CSE surface covered by GrO-IL nanocomposite (a), AuNPs-Chit bioconjugate (b), and hybrid GrO-IL-AuNPs-Chit nanocomposite (c).

In order to improve the electroanalytical properties of GrO, it was modified by a room temperature imidazolium ionic liquid ([BMIM]PF_6_) [49, 50]. The procedure for the preparation of the GrO-IL nanocomposite have been previously reported in [45]. GrO nanosheets (10 mg) dispersed in 2.0 mL of ethanol were mixed with the ionic liquid (50 *μ*L). Afterwards, the mixture was ultrasonically treated for 1 h, centrifuged, and dried to yield a black-brownish composite labelled as GrO-IL. Then, 10 mg of this composite was mixed with 1.0 mL of AuNPs-Chit bioconjugate solution by using ultrasonication for 30 min, generating the heterogeneous suspension labelled as GrO-IL-AuNPs-Chit.  Figure 2 displays the SEM images of GrO-IL, AuNPs-Chit, and hybrid GrO-IL-AuNPs-Chit (c) nanocomposites. The morphology of the hybrid nanocomposite indicated that it was relatively smooth and GrO-IL has been uniformly integrated with AuNPs-Chit.

Finally, assembly of the GO-IL-AuNPs-Chit-modified CSE was carried out as described previously [45]. Before modification, the surface of a pure СSE was mechanically polished with 0.3 and 0.05 *μ*m alumina powders. Then, the electrode was thoroughly cleaned with ethanol and deionized water, in order to remove residual alumina particles. The modification of the pretreated CSE was carried by drop casting 5 *μ*L of the GrO-IL-AuNPs-Chit suspension onto its surface followed by subsequent air drying at room temperature for 1 h. Then, in order to stabilize the formed layer, the resulting film was applied with 2 *μ*L of 0.02 mol·L^−1^ NaOH solution followed by a drying step for 24 h. The electrochemical activation of the fabricated sensor denoted as GrO-IL-AuNPs-Chit/CSE was performed with cyclic voltammetry technique (0.0 – +1.4; 50 mV·s^−1^, 20 cycles) in PBS (0.1 mol·L^−1^, pH 7.4). The CSEs modified with only GrO-Chit and GrO-IL-Chit were also prepared through similar procedure for comparison.

### 2.4. Preparation of DNA Samples

Thermally denatured ds-DNA was prepared according to the literature [51]. Ultrasonic irradiation of ds-DNA was carried out in a sonication bath in 0.1 mol·L^−1^ phosphate buffer solution (PBS, pH 7.4) for 15 min. Acidic denaturation of ds-DNA was done by treatment in 0.5 mol·L^−1^·НСlO_4_ as described early [52].

## 3. Results and Discussion

### 3.1. Comparative Electrochemical Characterisation of Various Decorated CSEs

**Figure 3:**
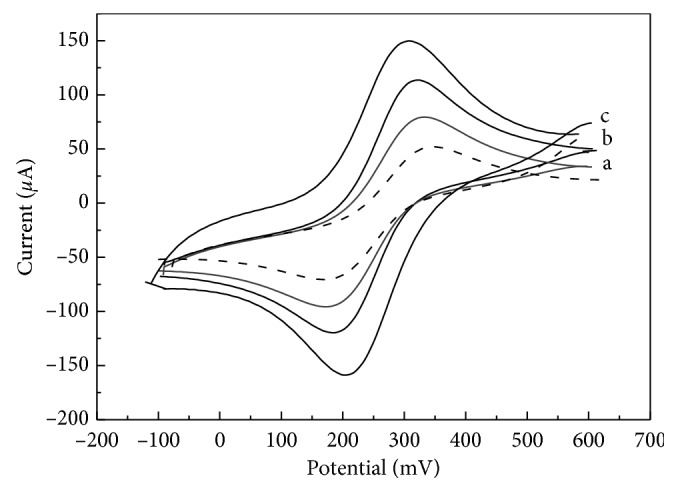
The CVs recorded at GrO-Chit/CSE (a), GrO-IL-Chit/CSE (b), and GrO-IL-AuNPs-Chit/CSE (c) in 5.0 mmol·L^−1^ K_3_[Fe(CN)_6_] containing 1.0 mol·L^−1^ KCl (*v* = 0.1 V·s^−1^). The voltammogram recorded at a bare CSE is indicated by a dotted line.

The prepared sensing nanomaterials were characterized by cyclic voltammetry (CV) in 5.0 mmol·L^−1^ K_3_[Fe(CN)_6_] solution containing 1.0 mol·L^−1^ KCl at a scan rate *v* from 0.01 to 0.30 V·s^−1^. As could be seen from the CVs presented in Figure 3, GrO-Chit/CSE (curve a) showed the smallest quasireversible voltammetric response and the peak-to-peak separation potential Δ*E*_p_=|*E*_pa_ − *E*_pc_|=160  mV.

In case of CSE covered with GrO-IL-Chit composite film, an obvious increase in the voltammetric response was observed (Figure 3(b)). It should be noted that intercalating IL in GrO can enhance the distance between the layers of GrO, resulting a higher electroactive surface area of the sensor (*A*_act_ = 0.144 ± 0.005 cm^2^) compared to pure GrO (*A*_act_ = 0.116 ± 0.003 cm^2^). After CSE modification with the GrO-IL-AuNPs-Chit composite film, the redox peak currents further increased and Δ*E*_p_ value obviously decreased up to 97 mV (Figure 3(c)). This may be due to the largest effective surface area of GrO-IL-AuNPs-Chit/CSE (*A*_act_ = 0.203 ± 0.005 cm^2^) as well as to the excellent electrocatalytic activity of AuNPs.

Thus, it may be concluded that the GrO-IL-AuNPs-Chit hybrid composite showed advantages for the application as the sensing support material for the sensor.

### 3.2. Voltammetric Behaviour and Quantitation of Thiopurines at GrO-IL-AuNPs-Chit/CSE

**Figure 4:**
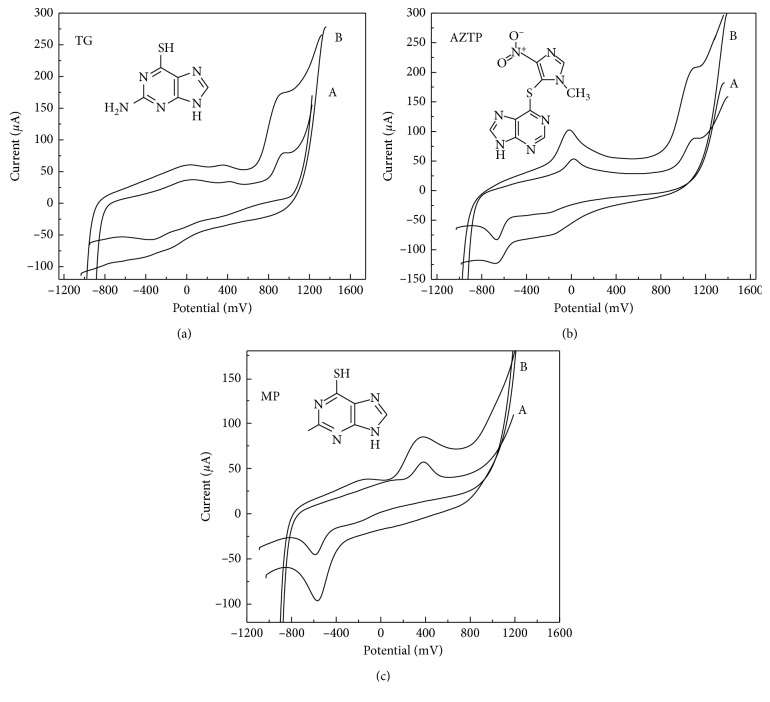
CVs recorded in 100 *μ*mol·L^−1^ solutions of TG, AZTP, and MP (PBS, pH 7.4; *v* = 0.1 V·s^−1^) at GrO-IL-Chit/GCE (A) and GrO-IL-AuNPs-Chit/CSE (B).

The CV behaviour of thiopurines at GrO-IL-AuNPs-Chit/CSE was investigated in 0.1 M PBS (pH 7.4) in comparison with GrO-IL-Chit/GCE. It is possible to see from Figure 4 that both sensors provided similar electrochemical behaviour. 6-TG exhibited only anodic peaks, indicating that its oxidation is an irreversible one. In contrast, the well-defined anodic and cathodic peaks were recorded for MP. AZTP, an imidazolyl derivative of MP, exhibited one additional oxidation peak at about 0 V, which can be related to the formation of nitroso derivative of AZTP [45]. The second anodic peak may be due to electrochemical reactions involving the imidazole ring oxidation, like in case of TG.

The presence of AuNPs in the new composite film highly promoted the voltammetric response toward all investigated compounds (Figure 4, curves B): the oxidation potentials shifted negatively with enhanced peak currents compared to GrO-IL-Chit/GCE (curves A). The above-described effects might be mainly explained not only to the synergistic effect of GrO-IL and AuNPs but also to the fact that thio-containing compounds are capable to bind to AuNPs-containing surfaces by formation of Au-S bonds.  Table 1 summarizes the analytical performance of the proposed sensor for the selected thiopurines.

The data show that GrO-IL-AuNPs-Chit/CSE provided very high detection sensitivity and wide linear ranges (two linear sections) with relatively low limits of detection (LODs). It characterized by good recoveries (Table 1) and storage stability for at least one month. Comparative evaluation of the developed sensor and Gr- (GrO-) based sensors found in the literature is given in Table 2. As it can be seen, the new sensor is characterized by higher sensitivity and a low detection limit for all three thiopurines.

### 3.3. Voltammetric Detection of ds-DNA at GrO-IL-AuNPs-Chit/CSE

**Figure 5:**
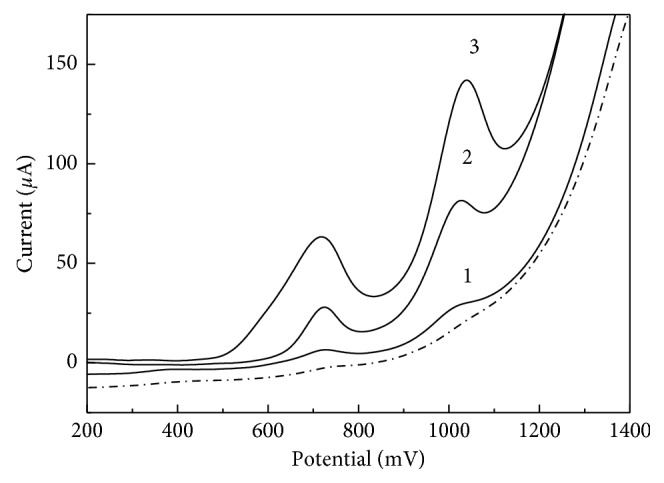
Adsorptive stripping voltammograms of thermally denatured ds-DNA (1), ultrasonically irradiated ds-DNA (2), and acid-treated ds-DNA (3) at GrO-IL-AuNPs-Chit/CSE after accumulation for 180 s under open circuit. The native DNA voltammogram is indicated by a dotted line.

It is well known that degradation of DNA in living organisms leads to mutations and the development of diseases. In this connection, evaluation of the intensity of this process is of great importance, in particular for environmental monitoring of genotoxic compounds [53]. Nanomaterial-modified electrodes can provide very simple and sensing platforms for DNA electroanalysis [54, 55]. The developed GrO-IL-AuNPs-Chit/CSE was found to have excellent adsorption ability and electrocatalytic activity towards the irreversible oxidation of the fish sperm ds-DNA in aqueous solutions (pH 7.4). Therefore, the given sensor was used to study degraded ds-DNA samples by means of adsorptive voltammetry approach. The accumulation of ds-DNA was performed in a stirred solution containing 10.0 *μ*g·mL^−1^ of the nucleic acid at open circuit potential for 180 s. After washing the electrode for 10 s with a buffer solution, the anodic voltammograms were recorded from +0.2 V to +1.4 V at the scan rate of 100 mV·s^−1^. As can be observed from Figure 5, the large difference in the oxidation signals is produced by the thermally degraded ds-DNA, ultrasonically irradiated ds-DNA, and acid treated ds-DNA samples. Voltammetric measurements in the solutions of both ultrasonically irradiated and acid-treated ds-DNA showed two well-defined oxidation peaks located around 0.7 V and 1.0 V (Figure 5, curves 2 and 3). These peaks can be attributed to the oxidation of DNA's purine bases (Gua and Ade)—residues of partial depurination of ds-DNA molecules. A noticeable decrease in anode peaks obtained in the thermally denatured ds-DNA solution could be explained by the inaccessibility of electroactive centers for the electron transfer. In this case ds-DNA acted like ss-DNA. The LOD for the thermally, ultrasonically, and perchloric acidic denatured ds-DNA was 0.5 *μ*g·mL^−1^, 0.3 *μ*g·mL^−1^, and 0.1 *μ*g·mL^−1^, respectively.

### 3.4. ds-DNA-MP Interaction Study

**Figure 6:**
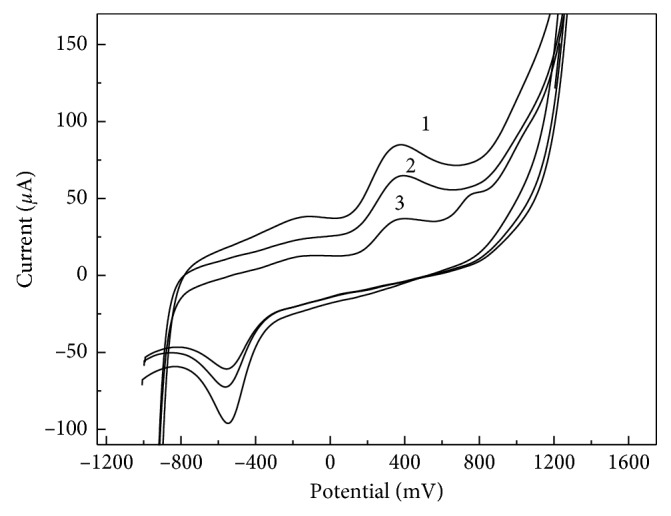
CVs recorded at GrO-IL-AuNPs-Chit/CSE during the interaction between MP and native ds-DNA. Interaction time in min: 3 (1), 10 (2), and 30 (3). Concentration of ds-DNA was 100 *μ*g·mL^−1^; the supporting electrolyte is 0.1 mol·L^−1^ PBS, pH 7.4; *v* = 0.1 V·s^−1^.

DNA is the pharmacological goal of many drugs. The interaction of DNA with small molecules represents a fundamental issue in life science and pharmaceutical screening, and it has been the subject of several investigations [55–58]. In order to investigate the possible interaction of the fish sperm ds-DNA with MP, GO-IL-AuNPs-Chit/CSE was immersed into 10 mL of the deoxygenated PBS (pH 7.4) containing 100 *μ*g·mL^−1^ MP and kept for 240 s under open circuit, for the MP immobilisation onto the electrode surface. Next, the interaction of MP with a 100 *μ*g·mL^−1^ solution of native ds-DNA (pH 7.4) was performed during different contact time periods ranging from 1 to 30 min at 37°C. The monitoring of the process was explored by measuring the changes of the MP voltammetric signals at −0.54 V and +0.38 V (the native ds-DNA was found to be electrochemically inactive in this potential range). As can be seen from Figure 6, the MP peak currents greatly decreased after the contact with ds-DNA. This effect can be corresponded to the probable intercalating of MP between purine base pairs of ds-DNA. The decrease in the MP signal was calculated as about 44% (*n*=5) by using 30 min of interaction time. The obtained results indicate that the interaction process of MP with ds-DNA is mainly the intercalation mode.

## 4. Conclusion

In this work, a novel electroactive material was proposed for the fabrication of a nonenzymatic electrochemical sensor. This material is a hybrid nanocomposite that combines a large GrO-IL surface area and highly conductive AuNPs stabilized with Chit functional groups. Cyclic voltammetric results confirmed that the developed sensor clearly exhibited the most electrochemical activity towards the electrooxidation of purine antimetabolites (6-thioguanine, 6-mercaptopurine, and azathioprine). The results demonstrated that it can be used for the determination of these compounds in a wide linear concentration range (up to 100–200 *μ*mol·L^−1^) with a high sensitivity (2.66–4.22 *μ*A/*μ*mol·L^−1^), a low detection limit (20–40 nmol·L^−1^, S/N = 3), and satisfactory recoveries (97.1–103.0%). Besides, the given sensor may be applied to study the double-stranded DNA damage and its interaction with anticancer drug 6-mercaptopurine in phosphate buffer solutions (pH 7.4) by using simple CV technique.

## Figures and Tables

**Table 1:** Main analytical results obtained for the voltammetric detection of the selected thiopurines using. GrO-IL-AuNPs-Chit/CSE (PBS, pH 7.4).

Analyte	*E*_p_, V	Regression equation Δ*I* (*I*_pa_ − *I*_blank_), *μ*A·vs. *c* (*μ*mol·L^−1^)	Linear range (*µ*mol·L^−1^)	LOD (*μ*mol·L^−1^) (S/N = 3)	Recovery, %
TG	+0.90 ± 0.02	Δ*I*^*∗*^ = 4.215*c* + 0.045 (*R*^2^ = 0.9989)	0–10	0.02	97.1–102.0
		Δ*I* = 1.528*c* + 0.083 (*R*^2^ = 0.9998)	10–150		99.7–101.5

AZTP	+1.11 ± 0.01	Δ*I*^*∗*^ = 2.660*c* + 0.056 (*R*^2^ = 0.9991)	0–10	0.04	96.5–103.0
		Δ*I* = 1.859*c* + 0.091 (*R*^2^ = 0.9991)	10–100		98.2–101.6

MP	−0.54 ± 0.01	Δ*I*^*∗*^ = 2.790*c* + 0.036 (*R*^2^ = 0.9990)	0–20	0.03	97.4–102.3
		Δ*I* = 0.938*c* + 0.065 (*R*^2^ = 0.9994)	20–200		99.5–100.7

^*∗*^Accumulation time (*t*_acc_): 120 s.

**Table 2:** Comparison of the Gr- (or GrO-) based sensors proposed for the determination of thiopurines by using adsorptive stripping voltammetry.

Compound	Sensor	Sensitivity (*μ*A/*μ*mol·L^−1^)	Linear range (*μ*mol·L^−1^)	LOD (*μ*mol·L^−1^)	*t*_acc_ (s)	Reference
AZTP	Gr-Chit/GCE	0.46	0.1–2.0	0.05	120	[39]
	Ag-Gr/GE	4.74	0.7–100	0.07	50	[40]
	GrO-IL-AuNPs-Chit/CSE	2.66	0.0–10	0.04	120	This work

TG	RGrO/CPE	0.23	0.4–50	0.07	40	[41]
	Poly(neutral red)-ERGrO/PGE	0.08	0.7–475	0.12	150	[42]
	GrO-IL-AuNPs-Chit/CSE	4.22	0.0–10	0.02	120	This work

MP	[Co(phen)_3_]^3+^/GrO-DNA/GCE	0.29	0.05–2.0	0.02		[43]
	GrO-IL-AuNPs-Chit/CSE	2.79	0.0–20	0.03	120	This work

GE: graphite electrode; GCE: glassy carbon electrode; CPE: carbon paste electrode; PGE: pencil graphite electrode.

## Data Availability

The data used to support the findings of this study are included within the article.
